# Hierarchical Micro/Nanostructures with Anti-Reflection and Superhydrophobicity on the Silicon Surface Fabricated by Femtosecond Laser

**DOI:** 10.3390/mi15111304

**Published:** 2024-10-27

**Authors:** Junyu Duan, Gui Long, Xu Xu, Weiming Liu, Chuankun Li, Liang Chen, Jianguo Zhang, Junfeng Xiao

**Affiliations:** 1 State Key Laboratory of Intelligent Manufacturing Equipment and Technology, School of Mechanical Science and Engineering, Huazhong University of Science and Technology, Wuhan 430074, China; duanjunyu@hust.edu.cn (J.D.); longgui@hust.edu.cn (G.L.); zhangjg@hust.edu.cn (J.Z.); 2 Hubei Jiuzhiyang Infrared System Co., Ltd., Wuhan 430223, China; 3 Wuhan National Laboratory for Optoelectronics, Huazhong Institute of Electro-Optics, Wuhan 430223, China; 4 China Ship Development and Design Center, Wuhan 430064, China; 13667160564@163.com (W.L.); ck_lee3376@163.com (C.L.); 5 State Key Laboratory of High-End Heavy-Load Robots, Midea Group, Foshan 528300, China; chenliangwy22@163.com; 6 Midea Corporate Research Center, Foshan 528311, China

**Keywords:** micro/nano structures, anti-reflection, superhydrophobicity, femtosecond laser, durability

## Abstract

In this paper, hierarchical micro/nano structures composed of periodic microstructures, laser-induced periodic surface structures (LIPSS), and nanoparticles were fabricated by femtosecond laser processing (LP). A layer of hydrophobic species was formed on the micro/nano structures through perfluorosilane modification (PM). The reflectivity and hydrophobicity’s influence mechanisms of structural height, duty cycle, and size are experimentally elucidated. The average reflectivity of the silicon surface in the visible light band is reduced to 3.0% under the optimal parameters, and the surface exhibits a large contact angle of 172.3 ± 0.8° and a low sliding angle of 4.2 ± 1.4°. Finally, the durability of the anti-reflection and superhydrophobicity is also confirmed. This study deepens our understanding of the principles of anti-reflection and superhydrophobicity and expands the design and preparation methods for self-cleaning and anti-reflective surfaces.

## 1. Introduction

As an abundant, environmentally friendly, and non-toxic semiconductor material, silicon is extensively utilized in various fields, including photodetection and photovoltaics [1,2,3]. However, its high reflectivity leads to substantial light loss [4]. Consequently, minimizing the reflection of incident light, enhancing the utilization rate of incident light, and increasing the photon absorption rate of silicon-based optical components have emerged as critical strategies for expanding and reinforcing the traditional applications of silicon devices. Traditional anti-reflective coatings, such as thin or multilayer films, have been widely used to achieve this goal [5,6,7]. However, most of these coatings are targeted at specific wavelength ranges and have poor durability. Therefore, the development of advanced micro/nano structures has emerged as a promising approach to enhance anti-reflective properties further [8,9,10]. It is possible to achieve significantly lower reflectance across a broad spectrum of wavelengths by leveraging the unique surface morphology and light-trapping mechanisms of micro/nano structures. These anti-reflective structures are often achieved by various methods, such as chemical etching [11], colloidal lithography and etching [12], and nanoimprinting [13]. It is worth noting that most reported methods require complex processes and typically carry a relatively high cost.

In addition, traditional anti-reflective surfaces are prone to the accumulation of dust, and the lack of self-cleaning functionality seriously affects their performance [14,15]. Superhydrophobic surfaces have garnered significant interest in recent years due to their unique functionalities and potential applications [16,17,18]. These surfaces exhibit remarkable water resistance, low adhesion of water droplets, and self-cleaning capabilities, making them ideal platforms for a wide range of practical applications [19]. The wettability of solid surfaces is determined by their structure morphology and chemical composition, while superhydrophobic surfaces are typically created by low surface energy substances and high roughness surfaces [16,20,21]. At present, the common methods for manufacturing superhydrophobic surfaces include chemical vapor deposition [22], chemical etching [23], laser processing [24], chemical reactions [25,26], etc.

The integration of superhydrophobic and anti-reflective features into a single surface presents a unique set of challenges and opportunities. The construction of micro/nano structures to achieve anti-reflection and superhydrophobicity simultaneously is a feasible method [27,28,29]. Laser processing has the advantages of high efficiency, low cost, and environmental friendliness among all manufacturing methods, and can easily fabricate micro/nano structures [30,31]. For example, L.B. Boinovich et al. fabricated a multimodal roughness with regular surface ripples on glass surface, and obtained a superhydrophobic surface with a contact angle of 160° and a rolling angle of less than 10° by adsorption of fluorosilane on the textured surface, but its optical performance changes have not been evaluated [32]. D.K. Chu et al. processed anti-reflection silicon with an excellent self-cleaning effect by combining an fs laser processing holes array with chemical modification [33]. The reflectance of the prepared surface was lower than 5%, and the surface exhibited a contact angle (CA) larger than 150° and a sliding angle (SA) lower than 3°. However, there is a lack of detailed research on the effects of different micro/nano structural morphologies, sizes, and chemical state changes on both the anti-reflective properties and hydrophobicity simultaneously [32,33,34,35,36]. It is possible to create structures that minimize light reflection while maximizing water resistance by carefully engineering the surface morphology and chemistry.

In this work, we fabricated hierarchical composite structures consisting of microstructure, LIPSS, and nanoparticles on silicon surfaces through simple single-step femtosecond laser processing. A layer of hydrophobic film formed on laser-ablated microstructures through perfluorosilane modification, obtaining superhydrophobic silicon surfaces with anti-reflection properties. By optimizing laser processing parameters, we explored and analyzed the effects of structural height, duty cycle, and size on reflectivity and superhydrophobicity. This work provides detailed analyses and insights into how laser processing parameters and chemical treatment affect these functionalities. Finally, after conducting a series of durability tests, it has been proven that the anti-reflective and self-cleaning surfaces have excellent stability to withstand external mechanical damage. The experimental results are significant for guiding researchers to efficiently and quickly prepare surfaces with durable superhydrophobicity and anti-reflection.

## 2. Experiments

### 2.1. Materials and Sample Preparation

Silicon wafer (30 × 30 × 5 mm^3^, the resistivity of 1~10 Ω·cm, n-type, double-sided polished) were purchased as a shelf product (Harbin Tebo Technology, Harbin, China). The samples were consequently ultrasonically cleansed with acetone, anhydrous ethanol, and deionized water for 10 min, then dried and stored for later use.

### 2.2. Laser Processing and Perfluorosilane Modification

A GS-FIR20 femtosecond laser system with 400 fs pulses at a central wavelength of 1030 nm and a repetition rate of 600 kHz was utilized for the fabrication of micro/nanostructured surfaces. The laser beam was focused on the surface of the silicon sample and scanned in a pattern of crossed lines in an atmospheric environment. Considering the acquisition of regular surface morphology, the laser power and scanning speed were set to 6 W and 600 mm/s, respectively, and the laser spot size was about 30 μm. The energy density was 1.415 J/cm^2^, and the number of pulses applied per spot was 30. The samples with different structural heights (H), duty cycles (D, defined as the ratio of the square of structural size to the square of the period size), and sizes (S) (Table 1; a detailed explanation of microstructure parameters can be found in Supplementary Figure S1a) were obtained by controlling the repeated number and scanning pitch. Then, all of the samples were washed with ethanol to remove processed dust and were slowly dried with pure nitrogen. At last, all the micro/nanostructured surfaces were immersed in the 1H, 1H, 2H, 2H-perfluorooctactyltrimethoxysilane alcohol solution with a concentration of 1% for 1 h, and then dried naturally. The laser processing schematic diagram, laser fabrication equipment, and laser processing parameters are shown in Supplementary Figures S1 and S2, and in Supplementary Table S1.

### 2.3. Characterization

The morphology of all the samples was examined by field-emission scanning electron microscopy (SEM, JSM-7600, JEOL, Tokyo, Japan) equipped with an energy-dispersive spectroscope (EDS, INCA-CH5, Oxford Instruments, Oxford, UK). The 3D topography measurement was characterized using a white-light interferometer (Zygo NewView 9000, ZYGO, CT, USA). The chemical composition of the samples was determined by X-ray photoelectron spectroscopy (XPS, AXIS-ULTRA DLD-600W, Shimadzu Kratos Corporation, kyoto, Japan).

A UV–Vis–NIR spectrophotometer (SolidSpec-3700, Shimadzu Corporation, kyoto, Japan) with an integrating sphere was utilized to investigate the light absorption behavior of every sample at 200–2500 nm. Contact angle (CA) and sliding angle (SA) measurements were carried out to evaluate the wettability of various surfaces by a video-based optical contact angle measuring device (OCA 20 from Data Physics Instruments). The droplets in this work are deionized water with a volume of 4 μL. The contact angles of every sample were examined at different randomly selected locations at least three times.

### 2.4. Durability Test

A scratch test was designed to evaluate the mechanical durability of the micro/nanostructured surfaces under severe abrasion conditions [37]. During the scratch test, a 1000-grit SiC sandpaper was selected as the abrasive surface. The micro/nanostructured surfaces were tested facing the abrasive surface with a fixed load of 200 g. The tape was stuck between the weight and the back of the sample with a linear speed of around 1 mm/s, which the sample and the weight can move together along with the dragging of the tape. The reflectivity and contact angle were recorded after 10 abrasion cycles. The schematic illustration of the experimental setup is shown in Supplementary Figure S3. The scotch tape test was used to evaluate the adherence performance of the as-fabricated superhydrophobic layer on the silicon substrate [38]. During the test, the scotch tape was pressed onto and peeled off from the sample surface, and the reflectivity and contact angle of the sample were tested after 10 peeling attempts. To verify the micro/nanostructured surfaces’ long-term performance, the reflectivity and contact angle were recorded after 30 days of exposure to the natural environment.

## 3. Results and Discussion

### 3.1. Fabrication and Characterization of Micro/Nanostructured Surfaces

**Figure 1:**
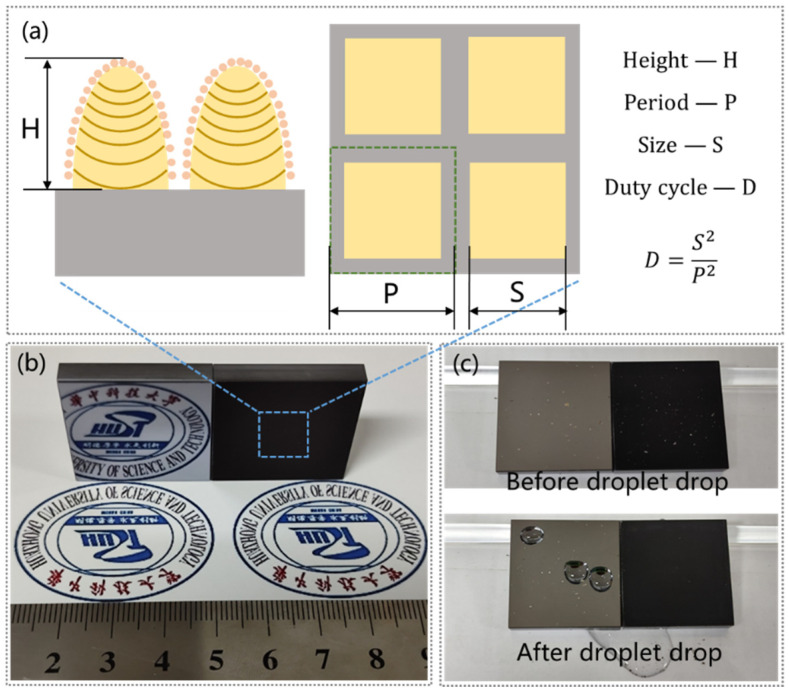
(**a**) Detailed explanation of microstructure parameters, (**b**) the anti-reflective effect of samples (The pattern is the emblem of Huazhong University of Science and Technology), and (**c**) the self-cleaning effect of samples before and after the droplet drop.

All micro/nanostructured surfaces were fabricated through single-step scanning by ultrafast laser processing. The typical machining time for a 30 × 30 mm sample, like the one in Figure 1b, was about 1.2 h. The height of different microstructures was controlled by the repeated number, and the duty cycle and size were determined by the scan pitch. The hierarchical morphology (Supplementary Figure S4) obtained by the white-light interferometer indicated that the designed micro/nanostructured surfaces had been prepared, and specific parameters are shown in Supplementary Table S2.

**Figure 2:**
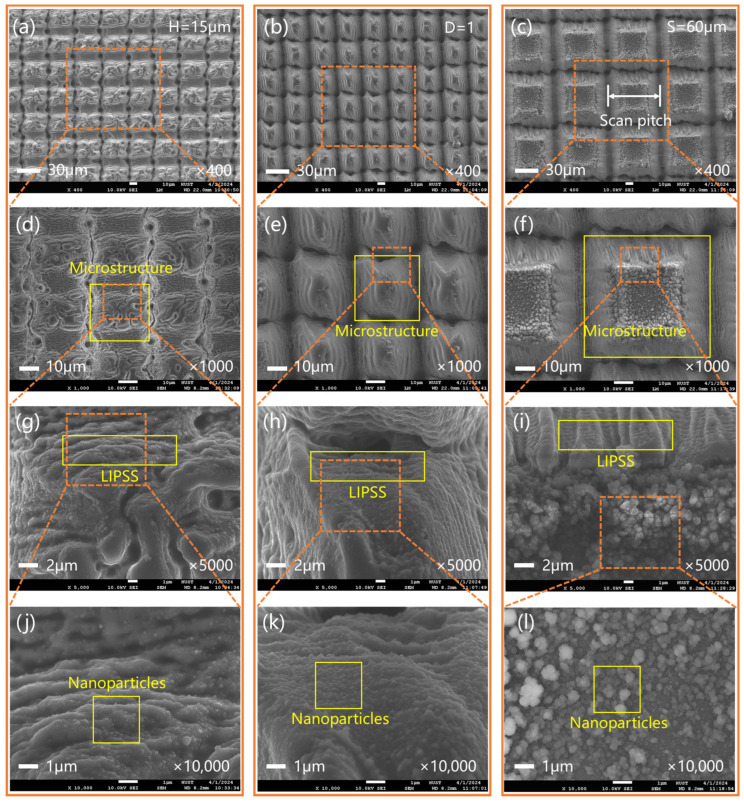
SEM images of micro/nanostructured surfaces. (**a**–**c**) Micro/nanostructures with different structural heights (H), duty cycles (D), and sizes (S). (**d**–**f**) Microstructures; (**g**–**i**) LIPSS; (**j**–**l**) nanoparticles.

The surface morphology of the micro/nano structures was observed by SEM, as shown in Figure 2. All micro/nanostructured surfaces were hierarchical composite structures consisting of a periodic microstructure, laser-induced periodic surface structures (LIPSS), and nanoparticles. The microstructure presented a regular arrangement, and its period was related to the scan pitch (Figure 2d–f). The LIPSS was spontaneously formed on the surface of monocrystalline silicon by laser pulses, and its period was ~1 μm (Figure 2g–i). Nanoparticles with a diameter range of 100–500 nm were attached to the microstructures (Figure 2j–l). Scan pitch and repeated number would affect the surface laser heat affected area, which could significantly determine the structure morphology. As the number of repetitions increased, the surface LIPSS became regular, and more nanoparticles formed, as shown in Figure 2a,b. When the scan space was set to 60 μm, most of the nanoparticles accumulated at the top of the microstructure, and their size increased (Figure 2b,c).

### 3.2. Chemical Changes After Laser Processing and Perfluorosilane Modification

**Figure 3:**
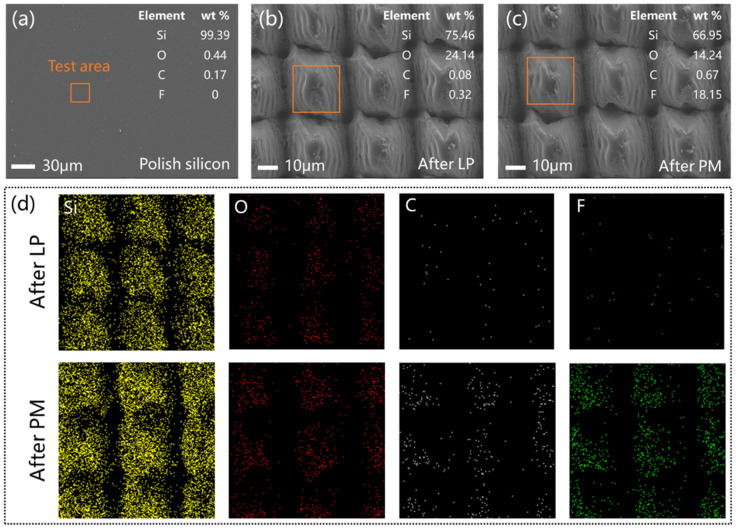
EDS results of micro/nanostructured surfaces. (**a**–**c**) Elemental content of the polished surface, after LP and after PM; (**d**) element distribution.

The elemental content of micro/nanostructured surfaces was analyzed qualitatively by EDS after laser processing (LP) and perfluorosilane modification (PM). After LP, the mass fraction of the surface oxygen element increased from 0.44% to 24.14% (Figure 3a,b). This indicates that laser processing caused oxidation reactions on the silicon surface. The mass fraction of fluorine element after LP was only 0.32%, but it increased to 18.15% after PM (Figure 3c). Due to the special structure and chemical properties of fluorosilane molecules, an extremely thin layer of fluorosilane molecular film was formed on the surface during the modification process, and the micro/nanostructure did not change. As shown in Figure 3d, the EDS surface scan obtained the element distribution information of the test area, and the fluorine element was uniformly distributed on the microstructure’s surface.

**Figure 4:**
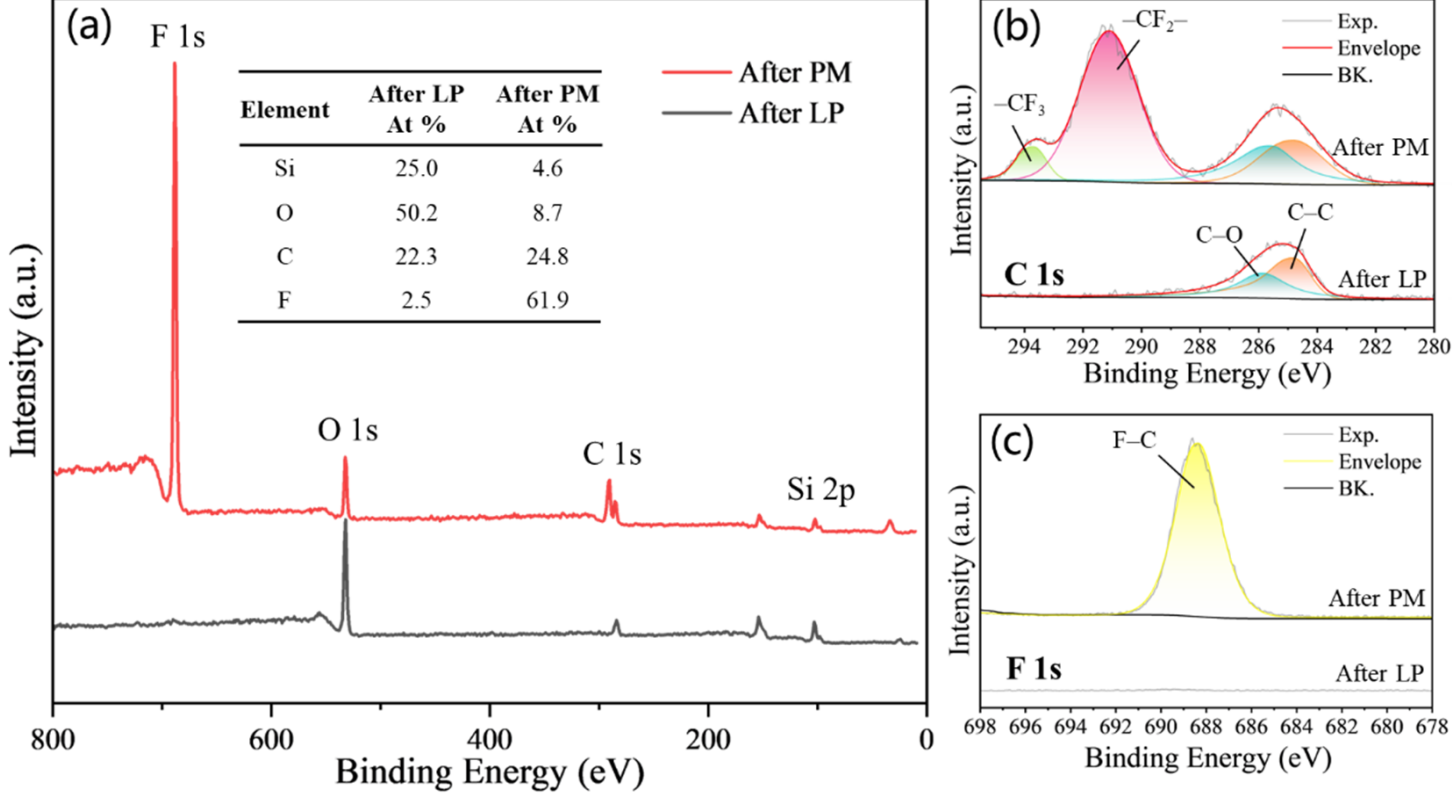
The XPS survey spectra of micro/nanostructured surfaces after LP and after PM. (**a**) XPS full spectrum. (**b**,**c**) High-resolution XPS spectra for C 1s and F 1s.

The chemical state of the surface element was analyzed qualitatively by XPS after LP and PM, as shown in Figure 4. The results of XPS show that the surface is mainly composed of Si, O, C, and F elements. After PM, the concentration of F atoms is 61.9%, which is 59.4% more than before. The composition of F increased remarkably, implying that the micro/nanostructured surface has been covered with fluoroalkyl molecular film. The high-resolution XPS spectra for C 1s (Figure 4b) show significant peaks at 291.3 eV and 293.7 eV, corresponding to the –CF_2_– peak and –CF_3_ peak, respectively [39]. From the F 1s high-resolution XPS spectra shown in Figure 4c, the main peak can be found at approximately 688.6 eV, and it is attributed to the F–C covalent bond [40]. These results further prove that fluoroalkyl is grafted on the micro/nanostructured surfaces, and the outermost surface is mainly composed of low-surface energy –CF_3_ and –CF_2_– components. As a result, the obtained surfaces were able to possess both micro/nano hierarchical structures and low surface energy at the same time, thus providing basic conditions for superhydrophobicity.

### 3.3. The Optical Property of the Micro/Nanostructured Surfaces

#### 3.3.1. The Anti-Reflective Principle of Micro/Nanostructures

Micro/nanostructured surfaces can effectively improve surface light absorption and exhibit significant anti-reflective properties. As shown in Figure 2, a micro/nano hierarchical composite structure was obtained by laser one-step preparation. This micro/nanostructure can utilize the multiple internal reflections of micrometer structures to achieve the effect of geometric “light trapping” [10,41], while the presence of subwavelength structures enhances the absorption of electromagnetic waves, thereby achieving anti-reflection effects [42].

**Figure 5:**
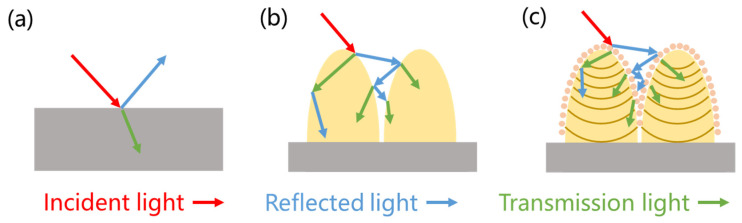
Schematic of the anti-reflective principle of light waves propagating through different characteristic structural surfaces. (**a**) Flat surface, (**b**) microstructures, and (**c**) micro/nanostructures.

Figure 5 shows a schematic of the anti-reflective principle of light waves propagating through different characteristic structural surfaces. When the incident surface is almost flat, a portion of the light is absorbed by the substrate material and another portion of the light is reflected out of the surface, which is related to the inherent characteristics of substrate material, as shown in Figure 5a. The schematic diagram of the propagation of light waves incident on the surface of microstructures (Figure 5b) indicates that when the size of the spacing between microstructures is much larger than the wavelength of the incident light, microstructures exhibit excellent light capture performance. Electromagnetic waves are captured and reflected multiple times in the microstructure, and an increase in optical path length leads to an increase in energy absorption. In addition, the presence of submicron and nanostructures with structural dimensions similar to or smaller than the wavelength further reduces the reflection phenomenon caused by the sharp change in refractive index, thereby reducing surface light reflection (Figure 5c). Therefore, the surface with micro/nanostructures is conducive to enhancing its surface anti-reflective properties.

#### 3.3.2. The Anti-Reflective Performance of the Micro/Nanostructured Surfaces

**Figure 6:**
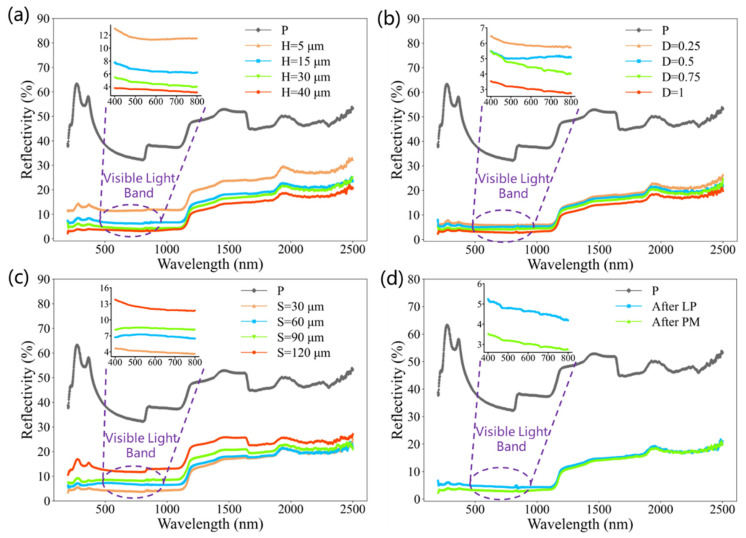
Reflectance spectra of micro/nanostructured surfaces at 200–2500 nm. (**a**) Different heights, (**b**) different duty cycles, and (**c**) different sizes. (**d**) After LP and after PM.

To determine the optimal anti-reflection effect, the surface reflectance spectra of micro/nanostructures with different heights, duty cycles, and sizes were compared in the range of 200–2500 nm. Figure 5 shows the reflectance spectra of micro/nanostructured surfaces at 200–2500 nm. All surfaces have a reduced reflectivity compared to the original polished silicon surface. The results of reflectivity with different heights (Figure 6a) show that higher structures have better anti-reflective properties because the number of light wave oscillations increases and the effective refractive index changes more gradually with an increase in the height of the structures. Especially in the visible light band, the average reflectance at 400–800 nm and at a height of 5 μm is 11.6%. As the height of the structure increases, the average reflectivity decreases to 3.5% when reaching a height of 40 μm, which is more than 90% lower than the reflectivity of 36.5% on polished silicon surfaces.

In Figure 6b, as the structural duty cycle increases, the surface reflectance shows a gradually decreasing trend. When the duty cycle increases from 0.25 to 1, the average reflectance of 400–800 nm decreases from 5.9% to 3.1%. An increase in the duty cycle means a decrease in the microstructure period, and there are more microstructure cavities per unit area to capture incident light, resulting in a decrease in surface reflectivity. The results in Figure 6c indicate that the size of the microstructure also has a significant impact on the surface reflectivity. The reflectivity of a structure with a size of 30 μm in the visible light range is only 4.0%, but as the structural size increases, the reflectivity continues to increase. Until the size increases to 120 μm, the average reflectivity increases to 12.2%, which is related to the fact that the increase in size causes more raw surfaces that have not been laser processed. Figure 6d shows the reflectivity after laser processing and perfluorosilane modification. After modification with perfluorosilane, the reflectivity of the sample decreased by 1.6% compared to the sample after laser processing. This is because fluoroalkyl silane has a very low refractive index (~1.3) at room temperature, much lower than the refractive index of the silicon substrate (~3.4), which can cause an obvious anti-reflective effect on the substrate surface.

Overall, the height, duty cycle, and size of microstructures are key factors affecting the surface reflectivity of micro/nanostructures, with the influence of height and size being more pronounced. The average lowest surface reflectivity in the visible light range is reduced to 3.0%, indicating that monocrystalline silicon has an excellent anti-reflection effect after preparing micro/nanostructures and fluorosilane molecular films. 

### 3.4. Wettability of the Micro/Nanostructured Surfaces

The wettability is mainly dependent on the surface microstructure and chemical composition of the material surfaces. The superhydrophobic surface requires low surface energy and needs micro/nanostructures to preserve the air, which suspends the droplets [16].

**Figure 7:**
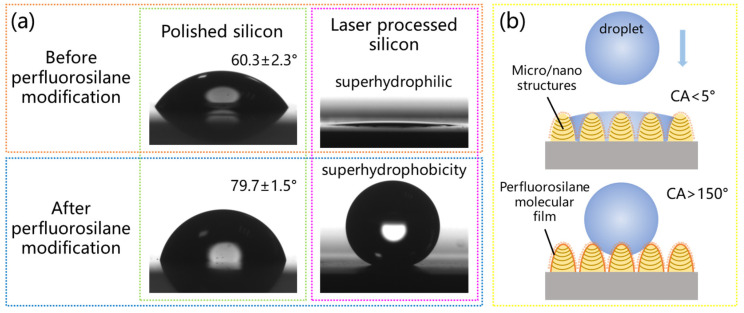
(**a**) The wettability of prepared surfaces before and after perfluorosilane modification. (**b**) Schematic diagram of wettability transition.

To evaluate the wettability of the multi-scale micro/nanostructured silicon surface, water contact angle (WCA) measurements were carried out. The water contact angle measurement of 60.3 ± 2.3° for the polished silicon surface is shown in Figure 7a. After laser processing, a large number of oxides are formed on the surface of silicon, and liquid droplets infiltrate into the microstructure according to the Wenzel formula [43], resulting in the surface of the micro/nanostructures exhibiting superhydrophilicity. To obtain hydrophobicity of the prepared surface, the typical fluoroalkyl silane modification is adopted here. After perfluorosilane modification, the surface treated with the laser changed from superhydrophilic to superhydrophobic. Rough structures can increase contact angles due to the decrease in the solid–liquid contact area according to the Cassie–Baxter formula [44], causing a superhydrophobic surface. Figure 7b shows a schematic diagram of the wettability transition. After the sample is immersed in the fluoroalkyl silane solution, hydrolysis and condensation reactions will occur, and the sample surface will self-assemble to form a single or multi-molecular film [29]. The outside of this film is covered by –CF3 and –CF2– groups, which have good hydrophobic properties [45]. In addition, the contact angle of the polished silicon surface after perfluorosilane modification only increased to 79.7 ± 1.5°, but the micro/nanostructured surface exhibits a large contact angle (CA) of 172.3 ± 0.8° and a low sliding angle (SA) of 4.2 ± 1.4° for water. This proves that both micro/nanostructures and low surface energy are indispensable conditions for the preparation of superhydrophobic surfaces.

**Figure 8:**
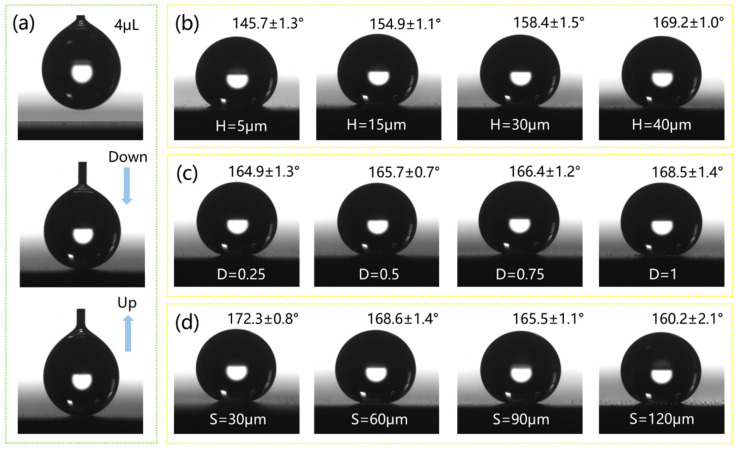
(**a**) The contact angle testing process of a 4 μL droplet. (**b**–**d**) The contact angle of micro/nanostructured surfaces with different heights, duty cycles, and sizes.

To obtain the trend of contact angle variances on micro/nanostructure surfaces with different heights, duty cycles, and sizes, the contact angles of all samples were measured, as shown in Figure 8. Figure 8a shows the contact angle measurement process of a 4 μL droplet. The results indicate that an increase in the height and duty cycle of the structure leads to the enhancement of surface hydrophobicity, and the smaller the structure size, the larger the contact angle. The change in contact angle can be explained by the Cassie model and the increased solid–liquid contact area [44]. This is consistent with the influence of structure on anti-reflection performance. Therefore, it can be concluded that a larger structural height, duty cycle, and smaller size corresponds to better anti-reflection and superhydrophobic performance. 

### 3.5. Durability of Anti-Reflection and Self-Cleaning Performance

**Figure 9:**
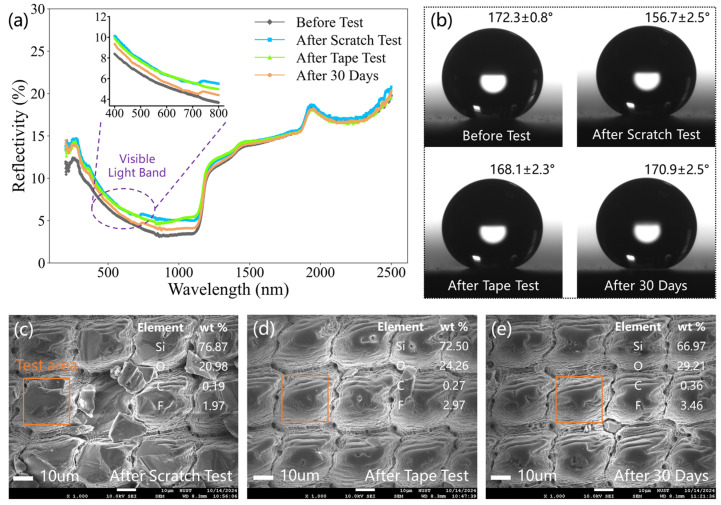
(**a**) Reflectance spectra of samples at 200–2500 nm after various tests. (**b**) The contact angle of samples after various tests. (**c**–**e**) Surface morphology and elemental content after scratch test, after tape test, and after 30 days.

Durability is a significant factor in practical engineering applications. In particular, physical and chemical damage can cause a serious loss of surface micro/nanostructure and chemical composition, which degrade the anti-reflection and super-hydrophobic properties. In this study, we used various methods to examine the durability of the micro/nanostructured surfaces, such as sandpaper abrasion, tape peeling, and long-term exposure to the natural environment. As shown in Figure 9, after various tests, the sample still maintains a low reflectivity and a contact angle exceeding 150°. After 30 days of exposure to the natural environment, SEM results (Figure 9e) showed that the micro/nanostructure did not change, but the surface oxidation increased the oxygen content, and the relative fluorine content decreased to 3.46%. The average reflectivity of the sample at 400–800 nm only increased from 5.4% to 6.0%, and the contact angle merely decreased by 1.4°. After ten cycles of powerful tape peeling, the fluoroalkyl molecular film was slightly damaged, and the fluorine content decreased to 2.97% (Figure 9d). However, the sample surface still exhibited excellent anti-reflection and superhydrophobic properties. Even after scratch tests, the surface structure and fluoroalkyl molecular film were damaged to a certain extent (Figure 9c). The average reflectivity of the sample at 400–800 nm was only 6.8%, and the contact angle was 156.7 ± 2.5°. These results demonstrate the mechanical stability and long-term anti-reflective superhydrophobic effect of the micro/nanostructured surfaces under various conditions. 

The anti-reflection and self-cleaning effects of micro/nanostructured surfaces are shown in Figure 1b,c. In contrast to the specular reflection of the original polished surface, the prepared sample exhibits no reflective images, highlighting its excellent anti-reflective performance. In practice, contaminants and dust adhering to a superhydrophobic surface are easily washed off by water, so the surface exhibits self-cleaning properties (See Movie S1).

## 4. Conclusions

In summary, we demonstrated a facile and simple approach to creating anti-reflection and self-cleaning silicon surfaces using laser processing and perfluorosilane modification. By adjusting the processing parameter, hierarchical micro/nanostructured surfaces with different heights, duty cycles, and sizes were obtained. The results indicate that surfaces with larger structural heights, duty cycles, and smaller structural dimensions have lower reflectivity and larger contact angles. The modification with perfluorosilane not only imparts low surface energy to the surface but also further reduces reflectivity. The average reflectivity of the silicon surface in the visible light band is reduced to 3.0% under the optimal parameters, and the surface exhibits a large CA of 172.3 ± 0.8 ° and a low SA of 4.2 ± 1.4°. Sandpaper abrasion, tape peeling, and exposure to natural environment tests show that the surface has excellent durability. This method could lead to applications in diverse areas including optical, detectors, and photovoltaics.

## Figures and Tables

**Table 1:** Parameters of all samples.

S/N	1	2	3	4	5	6	7	8	9	10	11	12
Height (μm)	5	15	30	40	25				30			
Duty cycle	1				0.25	0.5	0.75	1	1			
Size (μm)	30				30				30	60	90	120

## Data Availability

The original contributions presented in the study are included in the article/supplementary material, further inquiries can be directed to the corresponding authors.

## Supplementary Materials

The following supporting information can be downloaded at: https://www.mdpi.com/article/10.3390/mi15111304/s1.
