# Supplementary material for: Hierarchical Micro/Nanostructures with Anti-Reflection and Superhydrophobicity on the Silicon Surface Fabricated by Femtosecond Laser
Source: Micromachines (Basel). 2024 Oct 27;15(11):1304. doi: 10.3390/mi15111304 (PMC11596805; doi:10.3390/mi15111304)
Supplement: Supplementary file 1 [file micromachines-15-01304-s001.zip › Supplementary Information.pdf]

Supplementary Information

Hierarchical Micro/nano Structures with Anti-reflection and Superhydrophobicity on the Silicon Surface Fabricated by Femtosecond Laser

Junyu Duan[1], Gui Long[1], Xu Xu[2,3*], Weiming Liu[4], Chuankun Li[4], Jianguo Zhang[1], Jianfeng Xu[1], Junfeng Xiao[1*]

(1. State Key Laboratory of Intelligent Manufacturing Equipment and Technology, School of Mechanical Science and Engineering, Huazhong University of Science and Technology, Wuhan 430074, China;

2. Hubei Jiuzhiyang Infrared System Co. Ltd., Wuhan 430223, China;

3. Huazhong Institute of Electro-Optics, Wuhan National Laboratory for Optoelectronics, Wuhan 430223, China;

4. China ship development and design center, Wuhan 43006, China.

* Corresponding authors.

E-mail addresses: xiaojf@hust.edu.cn (J, X), ioexuxu@163.com (X, X).)

Supplementary Figures:

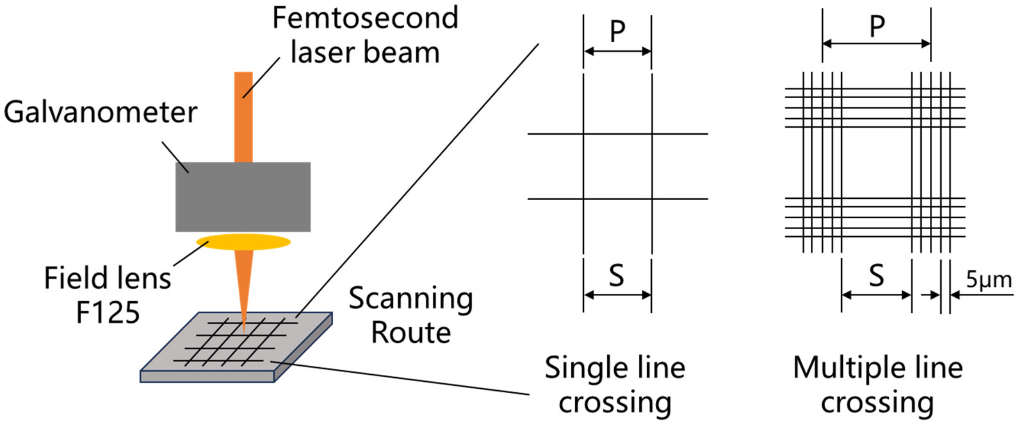

Figure S1. Schematic diagram and scanning route of femtosecond laser processing.

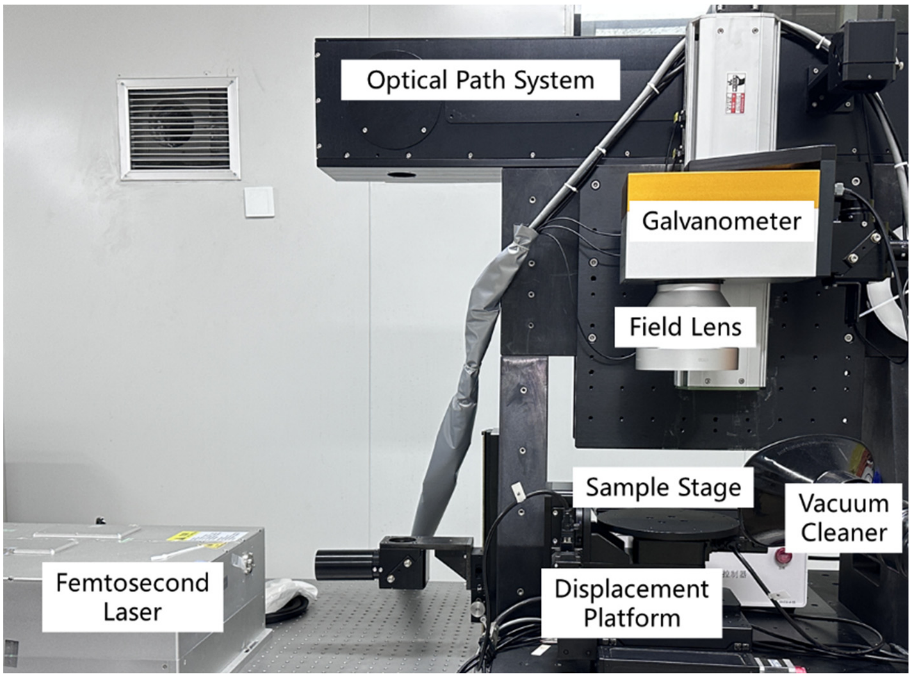

Figure S2. Laser fabrication equipment.

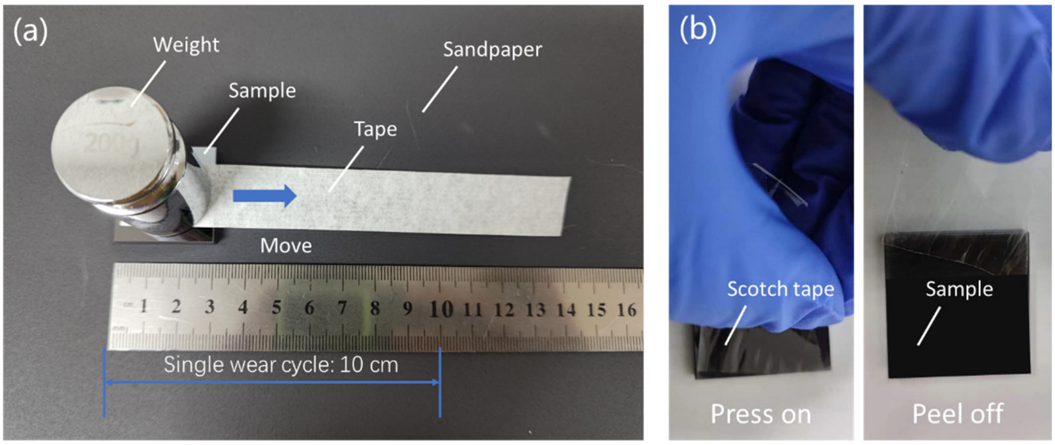

Figure S3. (a) Scratch test, (b) Scotch tape test.

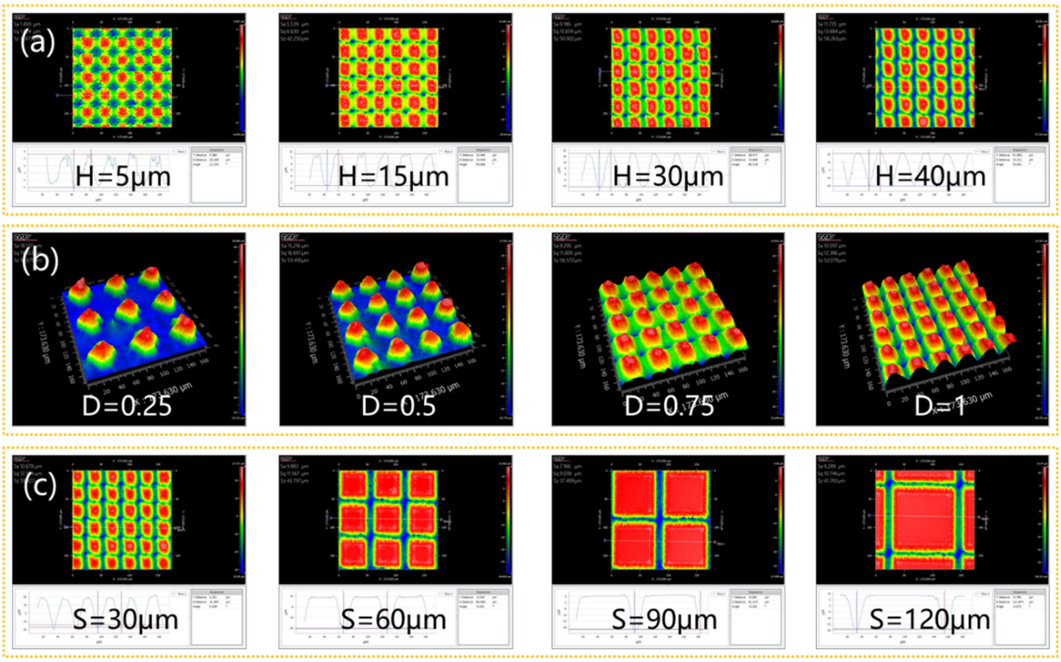

Figure S4. 3D topologies of different structural heights, duty cycles, and sizes.

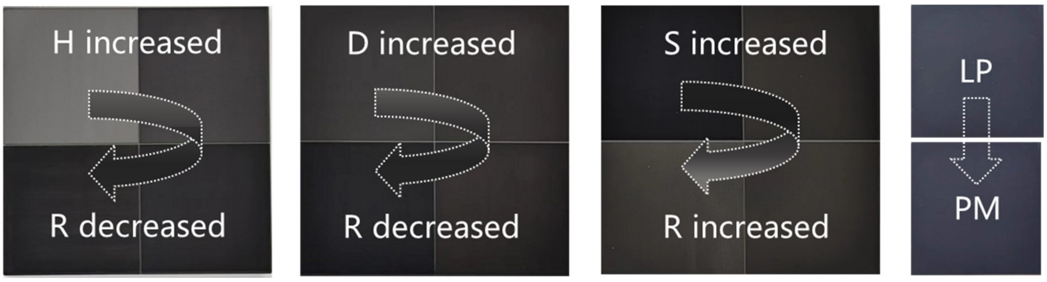

Figure S5. Micro/nanostructured surface images under natural light.

Supplementary Tables:

Table S1. Laser processing parameters for different structural heights, duty cycles, and sizes.

S/N	Laser Power (W)	Repetition rate	Scanning Speed (mm/s)	Scanning Route	Number of lines	Scanning Pitch (μm)	Repeated Number
1	6	600kHz	600	Single line crossing	1	30	3
2	6	600kHz	600	Single line crossing	1	30	11
3	6	600kHz	600	Single line crossing	1	30	22
4	6	600kHz	600	Single line crossing	1	30	30
5	6	600kHz	600	Multiple line crossing	6	30	5
6	6	600kHz	600	Multiple line crossing	4	30	9
7	6	600kHz	600	Multiple line crossing	2	30	13
8	6	600kHz	600	Multiple line crossing	1	30	20
9	6	600kHz	600	Single line crossing	1	30	25
10	6	600kHz	600	Single line crossing	1	60	25
11	6	600kHz	600	Single line crossing	1	90	25
12	6	600kHz	600	Single line crossing	1	120	25

Table S2. White light measurement results for different structural heights, duty cycles, and sizes.

S/N	Height (μm)	Size (μm)	Period (μm)
1	4.586	30.847	30.847
2	15.480	30.938	30.938
3	29.477	30.417	30.417
4	40.583	30.594	30.594
5	23.717	31.263	61.658
6	24.708	29.526	41.146
7	26.870	30.263	34.906
8	25.338	31.069	31.069
9	31.403	30.944	30.944
10	29.734	59.895	59.895
11	29.272	91.319	91.319
12	28.899	120.527	120.527
